# Wheat Straw Biochar Amendment Increases Salinity Stress Tolerance in Alfalfa Seedlings by Modulating Physiological and Biochemical Responses

**DOI:** 10.3390/plants14131954

**Published:** 2025-06-26

**Authors:** Shangzhi Zhong, Pengxin Hou, Congcong Zheng, Xuechen Yang, Qibo Tao, Juan Sun

**Affiliations:** 1 College of Grassland Science, Qingdao Agricultural University, Qingdao 266109, China; zhongsz@qau.edu.cn (S.Z.); hpx1031@163.com (P.H.); taoqibo@qau.edu.cn (Q.T.); 2 Shandong Key Laboratory for Germplasm Innovation of Saline-Alkaline Tolerant Grasses and Trees, College of Grassland Science, Qingdao Agricultural University, Qingdao 266109, China; 3 Institute of Computing Technology, Chinese Academy of Sciences, Beijing 100190, China; zhengcongcong@ict.ac.cn; 4 Institut National de la Recherche Agronomique, Unité Mixte de Recherche 1391, Interactions Sol-Plante-Atmosphère, 33140 Villenave d’Ornon, France; 5 State Key Laboratory of Ecological Safety and Sustainable Development in Arid Lands, Xinjiang Institute of Ecology and Geography, Chinese Academy of Sciences, Urumqi 830011, China; yangxuechen@ms.xjb.ac.cn

**Keywords:** *Medicago sativa*, gas exchange, osmotic adjustment, antioxidant enzyme, hormonal regulation

## Abstract

Salinity stress is a major environmental challenge that adversely impacts the physiological and biochemical processes of pasture, consequently resulting in reduced yields and compromised quality. Biochar amendment has recently emerged as a promising strategy to alleviate the deleterious effects of salinity stress. However, the interactive influences of salinity stress and wheat straw biochar on the physiological, biochemical, and growth characteristics of alfalfa (*Medicago sativa* L.) remain underexplored. A factorial experiment was conducted using a randomized complete design with five salinity levels (0, 25, 50, 75, and 100 mM NaCl) and three application rates of biochar (0, 25, and 50 g kg^−1^) to evaluate wheat straw biochar’s potential in alleviating salinity stress in alfalfa. Results showed that salinity stress increased oxidative stress (hydrogen peroxide and malondialdehyde) and reduced chlorophyll fluorescence (maximum quantum efficiency of photosystem II by 1–27%), leading to decreasing photosynthetic parameters, thereby constraining biomass accumulation by 9–77%. Wheat straw biochar amendment under the highest salinity stress, particularly at 25 g kg^−1^, mitigated oxidative stress by reducing H_2_O_2_ and MDA levels by 35% and 33%, respectively, while decreasing the antioxidant enzymes activities of CAT, POD, and SOD by 47%, 42%, and 39%, respectively, compared to the control (non-biochar addition). Concurrently, biochar restored the osmoregulatory substance concentrations of proline and soluble sugar by 59% and 33%, respectively, compared to the control. Furthermore, wheat straw biochar amendment increased the net CO_2_ assimilation rate by 98%, thereby increasing biomass by 63%. Our study demonstrates that wheat straw biochar can contribute to protecting alfalfa against salinity stress by modulating physiological and biochemical responses. These findings demonstrate that the 25 g kg^−1^ wheat straw biochar application had the best performance, suggesting this amendment could be a viable strategy for improving alfalfa productivity in salt-affected soils. Future research should explore long-term field applications and the underlying mechanisms of biochar–plant–soil–plant interactions under diverse saline-alkali environments.

## 1. Introduction

Soil salinity is a primary form of land degradation and a critical environmental bottleneck restricting global agricultural productivity [1,2,3]. Approximately 1.13 million hectares of worldwide land are affected by salinization, and this figure is projected to rise under the pressures of climate change, unsustainable irrigation practices, and intensive farming systems [4,5,6]. Salinity stress negatively impacts plants through a multifaceted mechanism involving nutritional imbalance, osmotic stress, and oxidative stress, ultimately disrupting physiological and biochemical processes and reducing crop yields [7,8,9,10]. Ion toxicity occurs when excessive sodium (Na^+^) and chloride (Cl^−^) ions in saline soils are absorbed by plants, inhibiting the uptake of nutrients (e.g., K^+^, Ca^2+^, and Mg^2+^), which has a cytotoxic effect on protein, nitrogen assimilation, and fat metabolism [11,12,13]. Concurrently, moderate and high soil salinity (EC > 4 dS m^−1^) lower water potential, creating an osmotic gradient that hinders plant water uptake and induces osmotic stress, which can impair photosynthetic capacity by disturbing leaf turgor, stomatal conductance, and chloroplast ultrastructure, ultimately reducing carbon fixation efficiency [1,14,15].

Furthermore, salinity stress promotes the excessive accumulation of reactive oxygen species (ROS), such as hydrogen peroxide (H_2_O_2_) and superoxide anions (O_2_^−^), leading to lipid peroxidation, oxidative damage to cellular organelles (e.g., mitochondria and chloroplasts), and disruption of thylakoid membrane integrity, thereby compromising photosystem II efficiency and electron transport chain function [16,17]. In parallel, salinity stress affects plant hormone metabolism by suppressing the biosynthesis of growth-promoting phytohormones, such as indoleacetic acid (IAA) and gibberellic acid (GA), while upregulating abscisic acid (ABA), thereby accelerating senescence and leaf abscission, and inhibiting meristematic activity [18,19]. Although plants activate adaptive responses such as stomatal closure, accumulation of osmolytes (e.g., proline and soluble sugar), and induction of antioxidant enzymes (e.g., catalase, peroxidase, and superoxide dismutase), these defense mechanisms may be insufficient to counteract severe or prolonged salinity stress, highlighting the need for agronomic interventions to enhance soil quality and plant tolerance [20,21,22].

Among various soil amendment technologies, biochar has emerged as a promising technology to enhance soil quality and improve plant tolerance to salinity stress [23,24,25,26,27]. Biochar is a carbon-rich, porous material produced by pyrolyzing organic biomass (e.g., agricultural waste, organic residues, and urban waste) at high temperature (between 300 °C and 1000 °C) in the absence or limited presence of oxygen conditions [28,29,30]. Its stable chemical structure, large specific surface area, and abundant functional groups endow biochar with unique properties, including improved soil cation exchange capacity (CEC), enhanced water holding capacity, and stimulation of soil microbial activity [31,32,33,34]. Mechanistically, biochar mitigates salinity stress through multiple pathways: (1) it enhances potassium (K^+^) uptake while reducing sodium (Na^+^) absorption by plants, thereby alleviating ion toxicity and restoring ionic homeostasis [35]; (2) its porous microstructure provides niches for beneficial soil microbiota, such as arbuscular mycorrhizal fungi (AMF) and plant growth-promoting rhizobacteria (PGPR), thereby fostering a resilient rhizosphere that facilitates nutrient acquisition under saline conditions [36,37]; and (3) biochar amendment modulates plant physiological responses by regulating phytohormone balance (e.g., a decreasing ABA levels), which may facilitate stomatal conductance and enhances water-use efficiency (WUE) by reducing excessive transpiration under salinity stress [18].

Despite its well-documented soil amelioration benefits, biochar application in saline-alkaline soils remains controversial [32,38]. The core debate stems from its inherent alkalinity, particularly when derived from lignocellulosic feedstocks. Under conditions of high salinity, this alkalinity has the potential to elevate the pH of the soil beyond the optimal range, thereby reducing the availability of micronutrients and disrupting the functions of root membrane transport [18,39]. Additionally, the efficacy of biochar is highly dependent on pyrolysis temperature and feedstock type: low-temperature biochar (300–500 °C) that is rich in organic functional groups may enhance nutrient retention, whereas high-temperature biochar (600–1000 °C) with greater graphitization may improve soil structure but lack sufficient cation exchange capacity [36,40,41]. Moreover, challenges related to economic viability, including high production costs and transportation burdens, restrict its large-scale adoption in resource-constrained agricultural systems [42,43]. These knowledge gaps and practical challenges underscore the need to determine optimal application rates and investigate their interactive effects with salinity on physiological and biochemical responses.

Alfalfa (*Medicago sativa* L.), a widely cultivated leguminous forage crop, is known for its high biomass yield, nutritional values, and adaptability to marginal environments [44,45]. As a nitrogen-fixing species with deep root systems, it plays a pivotal role in enhancing soil fertility through biological nitrogen fixation and improving soil structure via root penetration [46,47]. Although alfalfa exhibits moderate tolerance to salinity, previous studies have documented substantial declines in growth, photosynthetic efficiency, and nutrient uptake under severe saline conditions (EC > 6 dS m^−1^) [48,49]. These observations highlight the need for complementary strategies to mitigate salinity-induced limitations in alfalfa production. Thus, integrating biochar application with alfalfa cultivation may offer a viable strategy for restoring saline soils and enhancing forage productivity [43].

However, the physiological and biochemical mechanisms by which biochar alleviates salinity-induced stress in alfalfa remain poorly understood, particularly concerning dose-dependent responses. Meanwhile, both the high cost (e.g., production, transportation, and high-dose application rates) and pH value (e.g., deterioration of soil environment caused by high biochar dosages) remain the major challenges to the widespread use of biochar in areas affected by salinity [38,42,50,51]. Wheat is one of the major cereal crops in China, and improper management of wheat straw as agricultural waste threatens agricultural sustainability, particularly in regions affected by salt stress. Therefore, it will be advantageous for green and sustainable agriculture to have better knowledge of the physiological and biochemical benefits of wheat straw biochar addition for alfalfa development under salinity stress.

In this study, we systematically evaluated the effects of wheat straw biochar amendment on alfalfa seedlings under salinity stress, focusing on the growth, leaf gas exchange, chlorophyll fluorescence, oxidative stress indicators, osmolyte accumulation, antioxidant enzyme activities, and phytohormone profiles. We posited three hypotheses based on the existing literature and preliminary observations: (1) salinity stress would inhibit alfalfa growth and physiological performance in a dose-dependent manner; (2) moderate wheat straw biochar amendment would mitigate these negative effects by improving physiological and biochemical indices, thereby restoring growth and photosynthetic efficiency; (3) excessive wheat straw biochar application might counteract its benefits due to potential negative effects on soil chemistry. Our findings aim to provide insights into sustainable strategies for improving crop productivity in salt-affected soils and guiding the rational use of biochar in saline agriculture.

## 2. Materials and Methods

### 2.1. Study Site and Experimental Design

The experiments were conducted 5–12 August 2023 in a greenhouse of Qingdao Agricultural University (120°39′ E, 36°31′ N, 50 masl), located in Qingdao City, Shandong Province, China. The soil in this study was collected from the topsoil (0–25 cm depth) and the type of soil was loam soil, equivalent to typical Phaeozem in the World Reference Base (WRB) system at the Modern Agricultural Science and Technology Demonstration Park campus of Qingdao Agricultural University (120°04′ E, 36°26′ N, 37 masl), near Jiaozhou City, Shandong Province, China.

A full-factorial randomized design was employed, involving two factors: salinity stress (S) and biochar amendment (B). Given that wheat is the dominant crop in the experimental region and after comparing previous studies (Table S1), we selected wheat straw as the main raw material for biochar and determined three wheat straw biochar application rates (B0: 0 g kg^−1^ biochar, B1: 25 g kg^−1^ biochar, and B2: 50 g kg^−1^ biochar). Meanwhile, the experiment comprised five salinity stress levels (S0: 0 mM, S1: 25 mM, S2: 50 mM, S3: 75 mM, and S4: 100 mM NaCl), resulting in fifteen treatment combinations. Each combination was replicated ten times, yielding a total of 150 pots. The biochar material used in this study was wheat straw biochar produced by Henan Lize Environmental Protection Technology Co., Ltd. (Zhengzhou, China), at a temperature of 500 °C by carbonizing wheat straw under anaerobic conditions. Prior to the experiment, air-dried soil was passed through a 0.5 mm sieve. The wheat straw biochar at 0 g kg^−1^, 25 g kg^−1^, and 50 g kg^−1^ was then added to plastic pots (48 cm in diameter and 46 cm in height), each containing 10 kg of soil per pot and thoroughly mixed [44]. Prior to transplanting, biochar-amended soils were equilibrated for 14 days and pots were watered to approximately 70% of field capacity. The detailed basic physicochemical characteristics of the soil and wheat straw biochar used are presented in Table 1.

The alfalfa (*Medicago sativa* L.) variety cultivar was “Zhongmu No.3” from China. Seeds were surface-sterilized by 75% ethanol for 1 min, rinsed with deionized water for 5 min, and sown in seedling trays. Seedlings were transplanted into pre-designed pots at the three- to four-leaf stage, ensuring uniform size across treatments [44]. Salinity treatments were initiated 30 days after transplanting using irrigation water with NaCl concentrations of 0, 25, 50, 75, and 100 mM. To prevent immediate salinity stress damage to the alfalfa seedlings, the salt concentration was gradually increased from 25 mM, finally reaching the predetermined maximum doses over 7 days. Salinity application was performed by controlling the soil moisture and EC values in the form of irrigation water. Before transplanting, mineral fertilizers were applied at rates of 150 mg kg^−1^ N, 25 mg kg^−1^ P, and 100 mg kg^−1^ K to support alfalfa seedlings to grow normally throughout the experiment. To ensure plant growth was not limited by soil water deficit, the pots were watered to approximately 70% of field capacity. The plants were kept under controlled conditions in a greenhouse with 25/20 °C day/night temperature, and 65–70% relative humidity.

After 90 days of treatment, measurements of leaf gas exchange and chlorophyll fluorescence, and the collection of fresh leaf materials were conducted. Mature leaves were sampled between 9:00 am and 11:00 am for the measurements of chlorophyll content, osmotic stress solutes, osmotically active compounds, antioxidant enzyme activities, and phytohormone concentrations. The collected samples were immediately flash frozen in liquid nitrogen and temporarily stored in a deep freezer (−80 °C) until analysis.

### 2.2. Leaf Gas Exchange Measurements

Leaf gas exchange parameters, including net CO_2_ assimilation rate (*A*), stomatal conductance (*g*_s_), and transpiration rate (*E*) were measured from 9:00 am to 11:00 am using a Li-6800 portable photosynthesis system (Li-Cor Inc., Lincoln, NE, USA). Measurements were taken on the uppermost, fully expanded leaves of the alfalfa seedlings (five replicates per treatment). Environmental conditions inside the leaf chamber were set to a photosynthetic photon flux density (PPFD) of 1500 μmol m^−2^ s^−1^, air temperature of 25 °C, and CO_2_ concentration of 400 ppm [52]. Leaf-level intrinsic water-use efficiency (*W*_g_) and instantaneous water-use efficiency (*W*_i_) were calculated as the ratios of *A* to *g*_s_ and *A* to *E*, respectively.

### 2.3. Chlorophyll Fluorescence Measurements

Following leaf gas exchange measurements, a chlorophyll fluorescence parameter (maximum quantum efficiency of Photosystem II, F_V_/F_M_) was measured on the third fully expanded leaf from the apex of alfalfa seedlings using a Li-6800 portable photosynthesis system (Li-Cor Inc., Lincoln, NE, USA). Before measurement, seedlings were dark-adapted for 30 min in a light-tight chamber prior to being submitted to the chlorophyll fluorescence procedure. A measuring light of about 0.5 μmol photon m^−2^ s^−1^ was set at a frequency of 600 Hz to determine the background fluorescence signal (F_o_). Then, a saturating flash of about 10,000 μmol photon m^−2^ s^−1^ and a duration of 0.8 s was applied for the estimation of the maximum fluorescence (F_m_). Leaf photochemical efficiency (maximum quantum efficiency of Photosystem II) was calculated as: F_V_/F_M_ = (F_m_ − F_o_)/F_m_ [53].

### 2.4. Chlorophyll Content Measurements

Chlorophyll content parameters, including chlorophyll a (Chl a), Chlorophyll b (Chl b), and total chlorophyll (total Chl), were measured and calculated according to Hiscox and Israelstam (1979) [54]. Leaf samples (approximately 0.2 g fresh weight) were punched as 5 mm diameter discs from the mid-vein region of fully expanded leaves. These discs were immersed in 2 mL of 95% acetone and shaken for 3 min to facilitate complete pigment extraction. Finally, the mixture was spun in a centrifuge to separate the chlorophyll extract. Concentration of Chl a, Chl b, and total Chl were determined at absorbance wavelengths of 663 nm and 645 nm using a microplate reader (Infinite M Plex, Tecan Trading Co., Ltd., Salzburg, Austria).

Chlorophyll contents were calculated using the following equations [54]:

$$C_{Chl\;a}(mg/L)=12.27\times A_{663}-2.69\times A_{645}$$

$$C_{Chl\;b}(mg/L)=22.88\times A_{645}-4.68\times A_{663}$$

$$C_{total}(mg/L)=8.02\times A_{663}+20.29\times A_{645}$$

$$Chlorophyll\;X(mg/gFW)=\frac{C_{x}\times V}{W\times 1000}$$

where C_chl a_, C_chl b_, and C_total_ represent the concentration of Chl a, Chl b, and total Chl, respectively; C_X_ represents C_chl a_, C_chl b_, and C_total_, respectively; V represents the volume of the extraction solution; and W represents the fresh weight of the measured leaf sample.

### 2.5. Oxidative Stress Solutes Measurements

To determine Malondialdehyde (MDA) contents (mmol g^−1^ FW), leaf samples (0.5 g fresh weight) were homogenized in 5 mL of 5% trichloroacetic acid [55]. The homogenate was centrifuged at 1790× *g* for 10 min at 25 °C. The supernatant was added to 2-thiobarbituric acid (TBA), and the mixture was heated at 98 °C for 10 min, then cooled to room temperature. After a second centrifugation at 1790× *g* for 10 min, absorbance was measured at 532 nm to quantify MDA concentration, with background correction at 600 nm to eliminate nonspecific interference [56]. Hydrogen peroxide (H_2_O_2_) concentration was assayed according to Farhangi-Abriz and Torabian (2017) [57]. Approximately 1 g of fresh plant samples was homogenized in 5 mL of 0.1% trichloro-acetic acid (TCA), and centrifuged at 16,099× *g* for 15 min at 4 °C to pellet cellular debris. A 0.5 mL aliquot of the supernatant was mixed with 0.5 mL of 10 mM potassium phosphate buffer (pH 7.0) and 1 mL of 1 M potassium iodide (KI) solution. The absorbance reading was taken at 390 nm, and H_2_O_2_ content was calculated using a molar extinction coefficient specific to the reaction product.

### 2.6. Antioxidant Enzyme Activity Measurements

Antioxidant enzyme activities, including catalase (CAT, EC 1.11.1.6), peroxidase (POD, EC 1.11.1.7), and superoxide dismutase (SOD, EC 1.15.1.1), were determined using the following procedures [52,57]. Leaf samples (approximately 0.5 g fresh weight) for enzyme activity analysis were homogenized in an ice-cold mortar with 6 mL of ice-cold 50 mM sodium phosphate buffer (pH 7.0) containing 0.2 mM EDTA and 1% (*w*/*v*) polyvinylpyrrolidone (PVP) to prevent protein oxidation and stabilize enzymes. The homogenates were filtered through cheesecloth and centrifuged at 4 °C for 20 min at 15,000× *g*. The supernatant was collected and used for the assays of enzymatic activities. CAT and POD activities were measured using the method of Maehly and Chance (1954) [58], which was based on the decomposition of H_2_O_2_ as monitored by a decline in absorbance at 240 nm and the observation of absorbance increase caused by the colored compound at 470 nm, respectively. SOD activity was assayed according to Giannopolitis and Ries (1977) [59], based on its ability to inhibit the photochemical reduction of nitroblue tetrazolium (NBT). The protein contents of crude enzyme extracts were determined according to Bradford (1976) [60] using bovine serum albumin (BSA) as the standard to normalize enzyme activity to protein content.

### 2.7. Osmotically Active Solute Measurements

Osmotically active solutes, including leaf proline and soluble sugar, were quantified using the acid-ninhydrin method and microplate enzymatic assay, respectively [61,62]. The acid-ninhydrin reagent was prepared by dissolving 1.25 g of ninhydrin in 30 mL glacial-acetic acid and 20 mL of 6 M phosphoric acid under gentle heating and stirring until fully dissolved. Leaf samples (approximately 0.5 g fresh weight) were homogenized with 3% sulfosalicylic acid (10 mL) and the homogenate was clarified by centrifugation at 3500× *g* for 10 min. For proline determination, 2 mL of the supernatant was mixed with 2 mL of acid-ninhydrin reagent and glacial acetic acid. The mixture was incubated in a 100 °C oven for 1 h, and rapidly cooled in an ice bath to terminate the reaction. After extracting the chromophore with 4 mL of toluene, the absorbance was read at 520 nm using toluene for a blank. Proline concentration was calculated from a standard curve and expressed on a fresh-weight basis. Each sample was analyzed in duplicate, and mean values were used for subsequent analysis.

Briefly, soluble sugar was extracted by adding 2 mL of 80% (*v*/*v*) ethanol (EtOH) to 70 mg of powdered plant tissue, followed by heating in an 80 °C water bath for 15 min with occasional vertexing. The extraction was repeated three times, and the combined supernatants were adjusted to a final volume of 6 mL with 80% (*v*/*v*) EtOH. Initial glucose concentration was measured at 340 nm using a microplate reader (Infinite M Plex, Tecan Trading Co., Ltd., Salzburg, Austria) against a glucose standard curve. To quantify total reducing sugar, 10 μL of phosphoglucose isomerase (PGI, Sigma P9544-0.25 EU, Merck KGaA Co., Ltd., Darmstadt, Germany) was added to convert fructose to glucose, followed by 10 μL of invertase solution (Sigma I4504-83 EU, Merck KGaA Co., Ltd., Darmstadt, Germany) to facilitate the hydrolysis of sucrose. After a 60 min incubation at 30 °C, absorbance at 340 nm was measured again to determine the combined glucose concentration from initial glucose, fructose, and sucrose hydrolysis [62].

### 2.8. Phytohormone Measurements

Mature, fully expanded alfalfa leaves were also collected to determine leaf phytohormone concentrations, including indoleacetic acid (IAA_leaf_), gibberellic acid (GA_leaf_), and abscisic acid (ABA_leaf_). Samples were immediately wrapped in aluminum foil and flash-frozen in liquid nitrogen to arrest metabolic activity, then stored at −80 °C until analysis. Concentrations of IAA_leaf_, GA_leaf_, and ABA_leaf_ were quantified using commercial enzyme-linked immunosorbent assay (ELISA, Shanghai Jianglai Biotechnology Co., Ltd., Shanghai, China) kits, following the protocol established by [63].

### 2.9. Growth and Biomass Measurements

Five pots were randomly selected per treatment for biomass measurements. Before harvest, five alfalfa seedlings were randomly selected from each pot to measure plant height, stem diameter, and leaf area, respectively. Leaf area was quantified using an LA-S scanner and analyzed with WinRhizo software version LA-S (Regent Instruments Inc., Sainte-Foy, QC, Canada). For each treatment pot, aboveground plant materials (shoot biomass) were harvested by clipping at ground level and belowground plant materials (root biomass) were carefully extracted and washed until free of soil through 0.2 mm mesh sieves. All the plant materials were first blanched at 105 °C for 30 min to inactivate enzymes, then oven-dried at 65 °C until they reached constant weight. Total biomass was calculated as the sum of shoot biomass and root biomass. The root/shoot ratio was determined by dividing root biomass by shoot biomass.

### 2.10. Statistical Analysis

All statistical analyses were carried out using SPSS software version 22 (SPSS Inc., Chicago, IL, USA), and figures were generated with SigmaPlot version 12.5 (Systat Software Inc., San Jose, CA, USA). A two-way analysis of variance (ANOVA) was performed to evaluate the main effects of salinity stress (S) and biochar amendment treatments (B), as well as their interaction effects (S × B), on the growth and biomass parameters (plant height, stem diameter, leaf area; shoot, root, and total biomass; and root/shoot ratio), leaf gas exchange parameters (*A*, *g*_s_, *E*, *W*_g_, and *W*_i_), the chlorophyll fluorescence parameter (F_V_/F_M_), chlorophyll content (Chl a, Chl b, total Chl, and Chl a:b ratio), oxidative stress solutes (MDA and H_2_O_2_), osmotically active solutes (leaf proline and soluble sugar), antioxidant enzyme activity (CAT, POD, and SOD), and phytohormone contents (IAA_leaf_, GA_leaf_, and ABA_leaf_). A one-way ANOVA was conducted to evaluate the interaction effects on measured variables. Tukey’s multiple comparison test was employed for post hoc analysis to identify significant differences for all the treatments. Data are presented as arithmetic mean ± standard error (SE). Additionally, correlation analyses among all measured indicators were performed using the GGally and ggplot2 packages in R software (R version 4.2.2 R, Core Team, Vienna, Austria).

## 3. Results

### 3.1. Effects of Wheat Straw Biochar Addition on Plant Growth Parameters Under Salinity Stress

**Figure 1:**
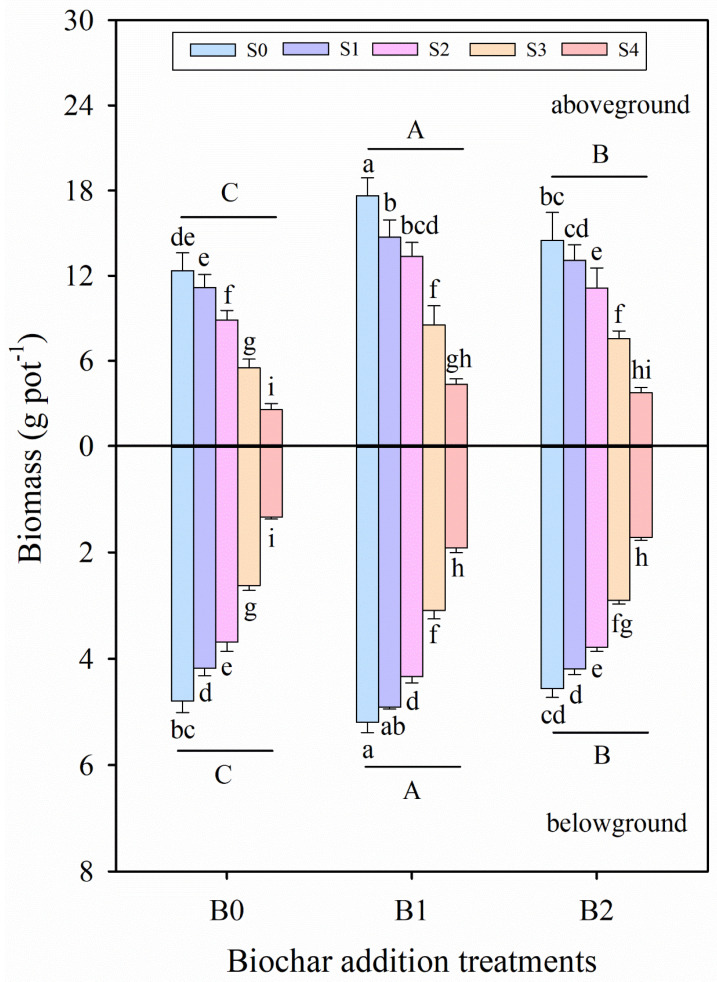
Shoot and root biomass of alfalfa seedlings under salinity stress and wheat straw biochar amendment treatments. Different capital letters indicate significant differences between measurement dates within the same wheat straw biochar treatment at the 0.05 level (*p* ≤ 0.05). Different lowercase letters indicate significant differences between salinity stress and wheat straw biochar amendment treatment combinations at the 0.05 level (*p* ≤ 0.05). S0, S1, S2, S3, and S4 refer to 0 mM, 25 mM, 50 mM, 75 mM, and 100 mM NaCl dose levels, respectively. B0, B1, and B2 refer to 0 g kg^−1^, 25 g kg^−1^, and 50 g kg^−1^ wheat straw biochar amendment levels, respectively. Data are presented as arithmetic mean ± standard error (n = 5).

The results obtained in the study indicate the effect of wheat straw biochar amendment on the growth parameters of alfalfa under salinity stress (Table 2 and Table 3; Figure 1). Compared with the control (S0B0 treatment), morphological traits and biomass indicators, such as plant height, stem diameter, leaf area, shoot biomass, root biomass, and total biomass, were significantly decreased with increasing salinity levels, except for the root/shoot ratio of alfalfa seedlings (*p* ≤ 0.001, Table 2). Salinity stress and biochar amendment treatments significantly affected all examined parameters (Table 2 and Table 3). Under the same salinity conditions, biochar amendment treatment (B1 and B2) enhanced the plant height by 9.31–55.89%, stem diameter by 2.72–16.28%, leaf area by 13.07–43.74%, shoot biomass by 17.14–73.84%, root biomass by 0.89–60.16%, and total biomass by 12.41–62.57% compared with no biochar addition treatment (B0). These results indicate that the application of biochar positively mitigated the adverse effects of salinity stress on plant growth. Among all treatments, the highest values of plant height, stem diameter, leaf area, shoot biomass, root biomass, and total biomass were observed under S0B1 treatment, whereas the lowest values were recorded in the S4B0 treatment. Additionally, the coefficients of variation for total biomass of alfalfa seedlings under B0, B1, and B2 treatments were 0.45, 0.38, and 0.40, respectively, across different salinity levels. Based on the average data of all growth parameters, the B1 treatment exhibited the significantly highest values for all the biochar addition treatments.

### 3.2. Effects of Wheat Straw Biochar Addition on Leaf Gas Exchange Parameters Under Salinity Stress

**Figure 2:**
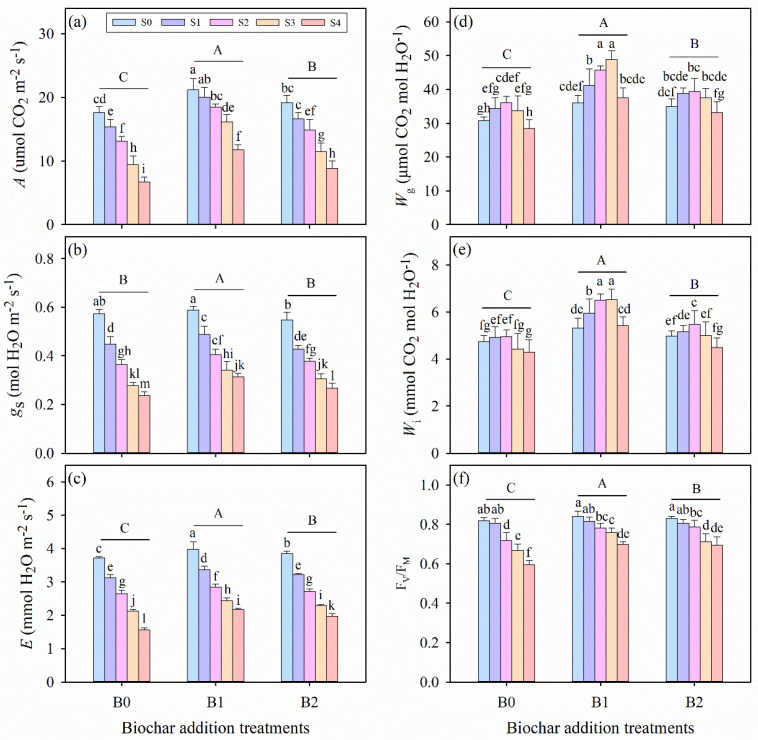
(**a**) Leaf net CO_2_ assimilation rate (*A*), (**b**) stomatal conductance (*g*_s_), (**c**) transpiration rate (*E*), (**d**) intrinsic water-use efficiency (*W*_g_), (**e**) instantaneous water-use efficiency (*W*_i_), and (**f**) maximum quantum efficiency of photosystem II (F_V_/F_M_) in alfalfa seedlings under salinity stress and wheat straw biochar amendment treatments. Different capital letters indicate significant differences (*p* ≤ 0.05) between measurement dates within the same wheat straw biochar treatment. Different lowercase letters indicate significant differences (*p* ≤ 0.05) between salinity stress and wheat straw biochar amendment treatment combinations. S0, S1, S2, S3, and S4 refer to 0 mM, 25 mM, 50 mM, 75 mM, and 100 mM NaCl dose levels, respectively. B0, B1, and B2 refer to 0 g kg^−1^, 25 g kg^−1^, and 50 g kg^−1^ wheat straw biochar amendment levels, respectively. Data are presented as arithmetic mean ± standard error (n = 5).

Salinity stress, biochar amendment, and their interaction significantly affected the net CO_2_ assimilation rate (*A*), stomatal conductance (*g*_s_), transpiration rate (*E*), intrinsic water-use efficiency (*W*_g_), and instantaneous water-use efficiency (*W*_i_) of alfalfa seedlings (*p* ≤ 0.01, Table 3). As salinity stress intensified, the values of *A*, *g*_s_, and *E* gradually decreased; however, biochar application mitigated this decline, increasing the values of these parameters (Figure 2a–c). The highest *A*, *g*_s_, and *E* values were observed in the S0B1 treatment, whereas the lowest values occurred under the highest salinity without biochar (S4B0 treatment). Notably, under B0, B1, and B2 treatments, *W*_g_ and *W*_i_ first increased to a maximum at specific salinity levels and then decreased as salinity further increased (Figure 2d,e). Biochar amendment enhanced both *W*_g_ and *W*_i_, with the B1 treatment exhibiting significantly higher values than B0 and B2 across all salinity levels. Compared with the B0 treatment, the B1 treatment enhanced *W*_g_ by 16.98%, 21.42%, 26.55%, 46.91%, and 33.29% under S0, S1, S2, S3, and S4 treatments, respectively. Similarly, *W*_i_ was increased by 12.77%, 22.50%, 31.76%, 51.56%, and 28.82% in the B1 treatment relative to the B0 treatment at the corresponding salinity levels.

### 3.3. Effects of Wheat Straw Biochar Addition on Chlorophyll Fluorescence Under Salinity Stress

Salinity stress, biochar amendment, and their interaction had significant impacts on the maximum photochemical efficiency of photosystem II (F_V_/F_M_) (*p* ≤ 0.01, Table 3). Across all biochar treatments, salinity stress significantly reduced F_V_/F_M_ (Figure 2f). Specifically, the data reveal that F_V_/F_M_ declined from 0.80, 0.84, and 0.83 in the S0 treatment to 0.60, 0.70, and 0.69 in the S4 treatment under B0, B1, and B2 treatments, respectively, which indicates that biochar addition has positively mitigated the adverse effects on chlorophyll fluorescence under salinity stress. Under the same salinity levels, the B1 treatment consistently exhibited significantly higher F_V_/F_M_ values than B0 and B2. Notably, no significant differences were observed between biochar treatments under moderate salinity conditions (*p* > 0.05, S1 and S2 treatments).

### 3.4. Effects of Wheat Straw Biochar Addition on Chlorophyll Content Under Salinity Stress

**Figure 3:**
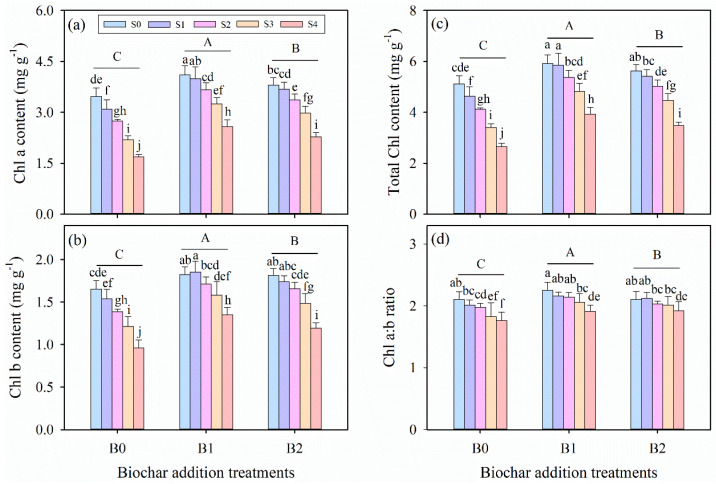
The contents of (**a**) chlorophyll a (Chl a), (**b**) chlorophyll b (Chl b), (**c**) total chlorophyll (total Chl), and (**d**) the ratio of chlorophyll a to chlorophyll b (Chl a:b ratio) in alfalfa leaves under salinity stress and wheat straw biochar amendment treatments. Different capital letters indicate significant differences (*p* ≤ 0.05) between measurement dates within the same wheat straw biochar treatment. Different lowercase letters indicate significant differences (*p* ≤ 0.05) between salinity stress and wheat straw biochar amendment treatment combinations. S0, S1, S2, S3, and S4 refer to 0 mM, 25 mM, 50 mM, 75 mM, and 100 mM NaCl dose levels, respectively. B0, B1, and B2 refer to 0 g kg^−1^, 25 g kg^−1^, and 50 g kg^−1^ wheat straw biochar amendment levels, respectively. Data are presented as arithmetic mean ± standard error (n = 5).

Two-way ANOVA tests indicated that the chlorophyll a (Chl a), chlorophyll b (Chl b), total chlorophyll (total Chl) content, and chlorophyll a:b ratio (Chl a:b ratio) were significantly affected by salinity stress and biochar amendment treatments (*p* ≤ 0.001, Table 3). For all biochar amendment treatments, Chl a content, total Chl content, and Chl a:b ratio decreased significantly with increasing salinity (*p* ≤ 0.05), while Chl b content showed a different pattern under the B1 treatment (*p* > 0.05, Figure 3c). Biochar amendment treatment increased the values of all the chlorophyll content parameters examined. Specifically, the B2 treatment exhibited the most pronounced increases in the aforementioned parameters across all salinity levels, outperforming both B0 and B1 treatments.

### 3.5. Effects of Wheat Straw Biochar Addition on Oxidative Stress Solutes Under Salinity Stress

**Figure 4:**
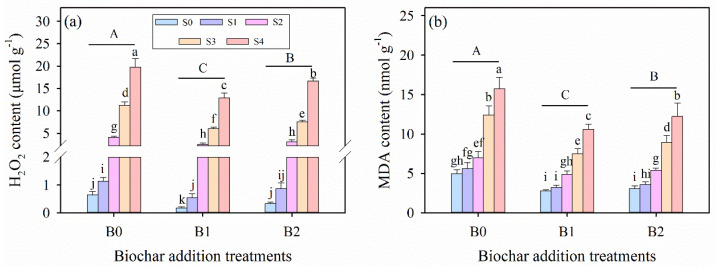
(**a**) Hydrogen peroxide (H_2_O_2_) and (**b**) malondialdehyde (MDA) contents in alfalfa leaves under salinity stress and wheat straw biochar amendment treatments. Different capital letters indicate significant differences (*p* ≤ 0.05) between measurement dates within the same wheat straw biochar treatment. Different lowercase letters indicate significant differences (*p* ≤ 0.05) between salinity stress and wheat straw biochar amendment treatment combinations. S0, S1, S2, S3, and S4 refer to 0 mM, 25 mM, 50 mM, 75 mM, and 100 mM NaCl dose levels, respectively. B0, B1, and B2 refer to 0 g kg^−1^, 25 g kg^−1^, and 50 g kg^−1^ wheat straw biochar amendment levels, respectively. Data are presented as arithmetic mean ± standard error (n = 5).

In parallel with the increasing salinity, statistically significant increases occurred in the oxidative stress solutes of malondialdehyde (MDA) and hydrogen peroxide (H_2_O_2_) of alfalfa seedlings for all biochar amendment treatments (Table 3; Figure 4). Additionally, salinity stress, biochar amendment treatments, and their interaction significantly affected the contents of MDA and H_2_O_2_ in alfalfa seedlings (*p* ≤ 0.001, Table 3). Meanwhile, the salinity-induced increases in these oxidative stress parameters were less pronounced in biochar-amended treatments compared to the no-biochar control (Figure 4). Comparing all the biochar amendment treatments, B1 treatment resulted in the lowest MDA and H_2_O_2_ under the same salinity levels, and the lowest values were both observed under S0B1 treatment (Figure 4). Under B1 treatment, compared with the S0B0 treatment, the contents of MDA and H_2_O_2_ in the S4B1 treatment were significantly increased by 1901% and 114%, respectively. Under B2 treatment, compared with the S0B0 treatment, the contents of MDA and H_2_O_2_ in the S4B2 treatment, were significantly increased by 2504% and 151%, respectively.

### 3.6. Effects of Wheat Straw Biochar Addition on Antioxidant Enzyme Activity Under Salinity Stress

**Figure 5:**
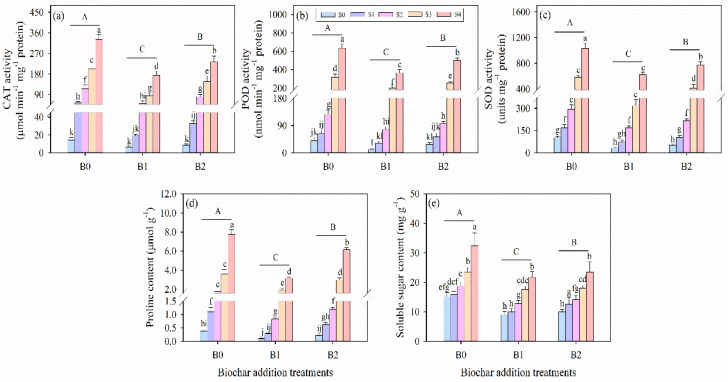
Leaf (**a**) catalase (CAT) activity, (**b**) peroxidase (POD) activity, (**c**) superoxide dismutase (SOD) activity, (**d**) proline content, and (**e**) soluble sugar content in alfalfa seedlings under salinity stress and wheat straw biochar amendment treatments. Different capital letters indicate significant differences between measurement dates within the same wheat straw biochar treatment at the 0.05 level (*p* < 0.05). Different lowercase letters indicate significant differences between salinity stress and wheat straw biochar amendment treatment combinations at the 0.05 level (*p* ≤ 0.05). S0, S1, S2, S3, and S4 refer to 0 mM, 25 mM, 50 mM, 75 mM, and 100 mM NaCl dose levels, respectively. B0, B1, and B2 refer to 0 g kg^−1^, 25 g kg^−1^, and 50 g kg^−1^ wheat straw biochar amendment levels, respectively. Data are presented as arithmetic mean ± standard error (n = 5).

Salinity stress and biochar amendment significantly influenced the antioxidant enzyme activities of catalase (CAT), peroxidase (POD), and superoxide dismutase (SOD) in alfalfa seedlings (Table 3; Figure 5a–c). Compared with the non-saline control (S0 treatment), the activities of CAT, POD, and SOD significantly increased under different salinity conditions (S1, S2, S3, and S4 treatments). However, both levels of biochar addition were recorded as reducing the activities of antioxidant enzymes in alfalfa seedlings under salinity stress. Comparing the same salinity stress treatments, these values of CAT, POD, and SOD were determined to be the lowest under B1 treatment compared to B0 and B2 treatments. Compared to the non-biochar addition at the 100 mM salinity level, B1 treatment decreased the antioxidant enzyme activities of CAT, POD, and SOD by 47, 42%, and 39%, respectively.

### 3.7. Effects of Wheat Straw Biochar Addition on Osmotically Active Solutes Under Salinity Stress

Osmotically active solutes, including proline and soluble sugar, increased significantly with escalating salinity stress intensity, whereas the application of biochar reduced their contents under the same salinity levels (Figure 5d,e). Salinity stress and biochar amendment significantly influenced the contents of proline and soluble sugar in alfalfa seedlings (*p* ≤ 0.001, Table 3). Specifically, the lowest proline and soluble carbohydrate levels were observed in the non-saline biochar-amended treatment (S0B1 treatment), which exhibited significant decreases of 75% and 40%, respectively, compared to the non-saline control without biochar (S0B0 treatment). Conversely, the highest level of these solutes was observed in the B0S4 treatment, recording a significant increase of 717% for proline and 44% for soluble sugar relative to the B0S0 treatment.

### 3.8. Effects of Wheat Straw Biochar Addition on Phytohormone Under Salinity Stress

**Figure 6:**
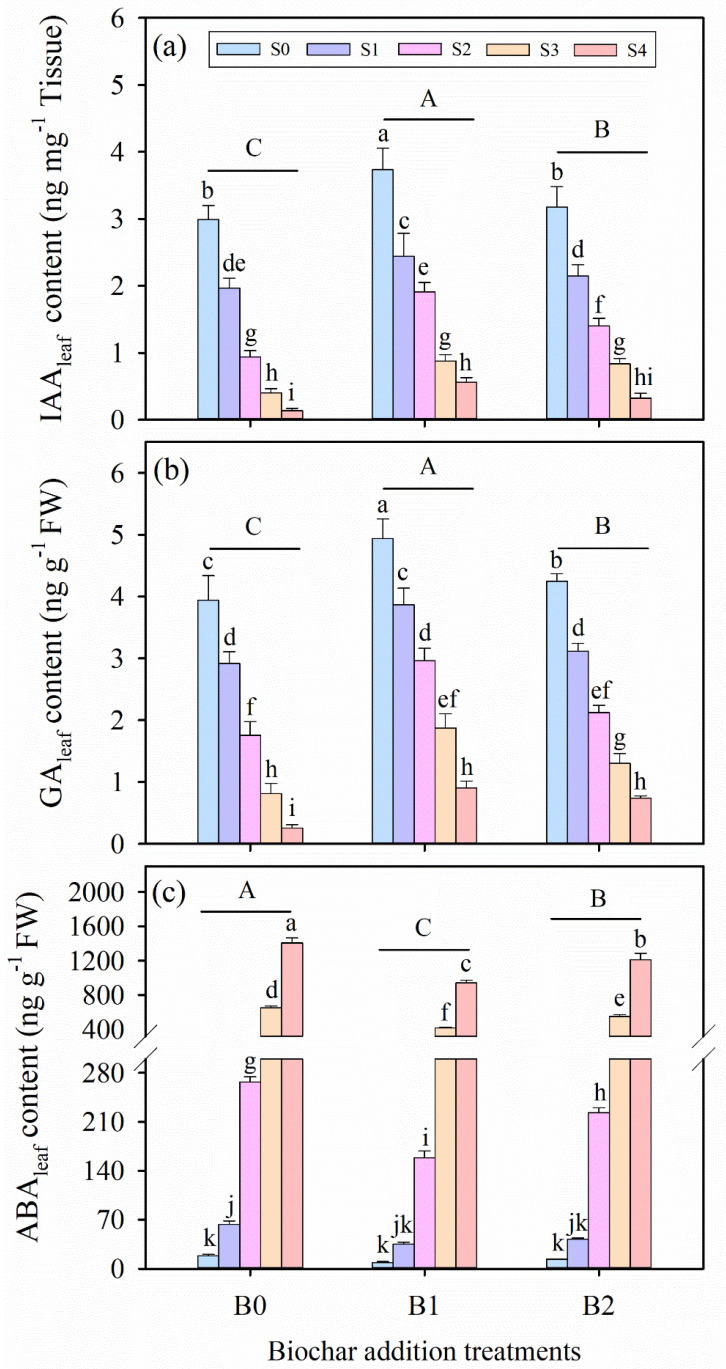
The contents of (**a**) indoleacetic acid (IAA_leaf_), (**b**) gibberellic acid (GA_leaf_), and (**c**) abscisic acid (ABA_leaf_) in alfalfa leaves under salinity stress and wheat straw biochar amendment treatments. Different capital letters indicate significant differences (*p* < 0.05) between measurement dates within the same wheat straw biochar treatment. Different lowercase letters indicate significant differences (*p* ≤ 0.05) between salinity stress and wheat straw biochar amendment treatment combinations. S0, S1, S2, S3, and S4 refer to 0 mM, 25 mM, 50 mM, 75 mM, and 100 mM NaCl dose levels, respectively. B0, B1, and B2 refer to 0 g kg^−1^, 25 g kg^−1^, and 50 g kg^−1^ wheat straw biochar amendment levels, respectively. Data are presented as arithmetic mean ± standard error (n = 5).

In our experiments, salinity stress, biochar amendment, and their interactions had significant effects on the contents of indole-3-acetic acid (IAA_leaf_), gibberellic acid (GA_leaf_), and abscisic acid (ABA_leaf_) in alfalfa leaves (*p* ≤ 0.01, Table 3). Salinity stress resulted in reductions in the IAA_leaf_ and GA_leaf_ contents of alfalfa leaves, but resulted in an elevation in ABA_leaf_ content (Figure 6). In addition, biochar amendment showed a tendency to increase the contents of IAA_leaf_ and GA_leaf_, while decreasing ABA_leaf_ content compared to CK treatment under salinity stress. Based on average data across all treatments, the B1 treatment exhibited the highest IAA_leaf_ and GA_leaf_ contents among the biochar addition treatments. Conversely, the B1 treatment consistently showed the lowest ABA_leaf_ contents under the same salinity conditions.

### 3.9. The Correlation Between Different Physiological, Biochemical, and Plant Traits

**Figure 7:**
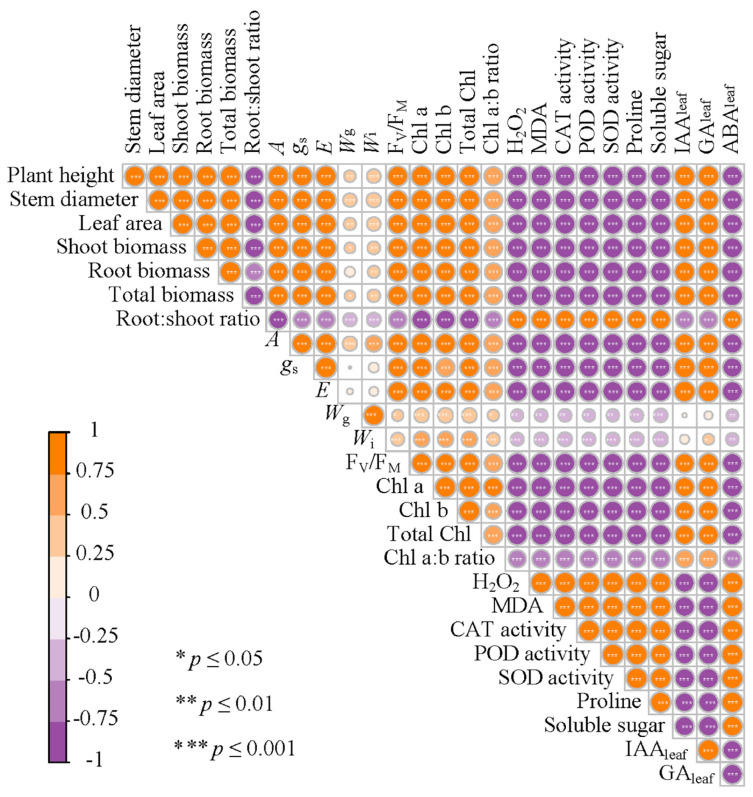
Correlation plots between all measured variables in the experiment. Color intensity and circle size represent the absolute value of the Pearson correlation coefficient.

**Figure 8:**
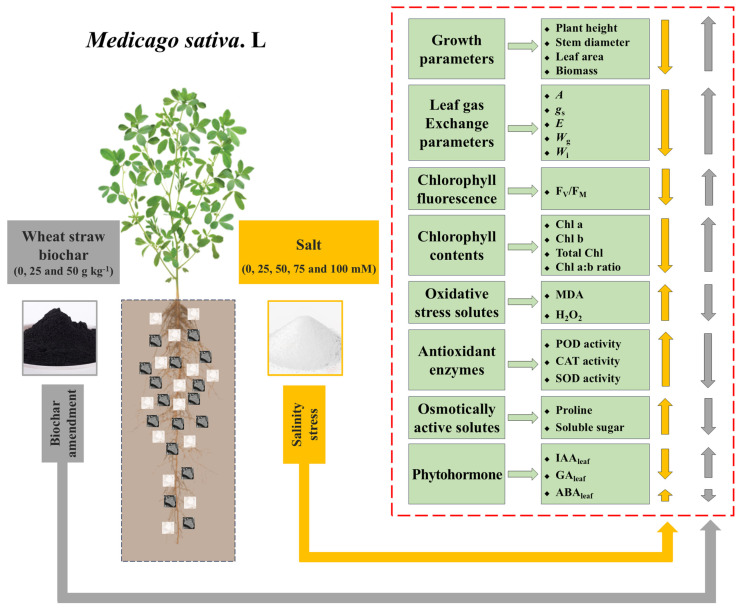
Schematic representation of wheat straw biochar amendment on the growth and physiological adaptations of alfalfa (*Medicago sativa* L.) under salinity stress, based on the findings of this study.

We further investigated the influence of wheat straw biochar on plant growth by assessing changes in physiological and biochemical indicators (Figure 7). According to the correlation study, the total biomass of *M. sativa* was positively associated with *A*, F_V_/F_M_, total Chl content, Chl a:b ratio, IAA_leaf_, and GA_leaf_, yet negatively with ABA_leaf_. Furthermore, *A* was also positively associated with F_V_/F_M_, Chl a content, Chl b content, total Chl content, and Chl a:b ratio. IAA_leaf_ and GA_leaf_ levels were negatively correlated with oxidative stress markers (H_2_O_2_ and MDA), while ABA_leaf_ levels showed a positive correlation. Based on the above analysis, it is hypothesized that wheat straw biochar amendment could promote the growth of *M. sativa* under both stress and non-stress conditions by improving multiple physiological and biochemical attributes (Figure 8).

## 4. Discussion

In this study, we evaluated the growth parameters of alfalfa seedlings and the photosynthetic and biochemical indices of their leaves under different salt stress and three wheat straw biochar treatments (0 g kg^−1^, 25 g kg^−1^, and 50 g kg^−1^). The objective was to investigate the potential effects of wheat straw biochar amendment and identify the optimal concentration for enhancing the stress-mitigation performance of alfalfa seedlings under salt conditions.

### 4.1. Effects of Wheat Straw Biochar Amendment on the Growth and Photosynthetic Characteristics of Alfalfa Under Salinity Stress

Plant morphological traits, such as plant height, leaf area, and shoot biomass accumulation, are highly sensitive to the salinity stress of alfalfa [2,3,10]. In this study, increased salinity significantly inhibited both aboveground and belowground growth of alfalfa seedlings (Figure 1). However, the application of biochar alleviated these negative effects, particularly enhancing plant height, leaf area, and biomass accumulation in both shoots and roots under saline conditions (Table 2 and Figure 1); these findings were in line with the study conducted by [64]. Additionally, there was a greater increase in the root-to-shoot ratio of alfalfa under the high-salinity treatments (S3 and S4 treatments) than under the low-salinity treatments (S1 and S2 treatments) (Table 2), which indicates that plants could adjust their biomass allocation strategies to adapt to severe abiotic stress [8,65]. This finding is in agreement with Zhong et al. (2024) [44], who observed that the trade-off of biomass allocation between aboveground and belowground tissues could optimize the resource acquisition capacity, and that biochar amendment could ensure absorption of water and nutrients and increase acquisition of light to enhance photosynthetic carbon fixation, thereby counteracting the growth stress of alfalfa under salinity conditions. The reason for the regulation of biomass allocation may be that the characteristics of biochar are low bulk density and large specific surface area, which could improve soil porosity and water holding capacity in the saline-alkali soil [18,32,33]. Additionally, numerous studies have reported that application of biochar increases the fertility of soils and the nutrient uptake of crops; this phenomenon can be explained by biochar itself having a large amount of available nutrients, which can be used as a nutrient supply in the soil [34,36,38,66]. Notably, application of wheat straw biochar significantly improved the water holding capacity and physicochemical properties of amended soils (Table S2 and Figure S1), suggesting that such amendment enhances alfalfa growth by improving the soil’s physicochemical properties.

Photosynthetic performance is one of the core physiological processes that is affected most by salinity, which limits the growth and development of numerous crops in saline-alkali soil [17,18,20]. In this study, we found that salinity stress significantly downregulated photosynthesis parameters in alfalfa leaves (Figure 2a–c), consistent with findings from most prior investigations [67,68,69,70,71]. Biochar application notably improved these parameters (Figure 2), demonstrating that biochar amendment mitigates photosynthetic inhibition under salinity stress [17,72]. The enhancement of photosynthesis in crops via biochar under saline conditions has also been reported by Yan et al. (2024) [73]. This promotion may involve two physiological mechanisms: (i) improvement of soil physicochemical properties and elevation of available water content [23,24,34], and (ii) provision of essential nutrients and enhancement of soil fertility in saline environments [26,50,74]. Additionally, variations in the degree of photosynthetic decline under different salinity levels may stem from stomatal dysfunction (stomatal limitation) or impairments to photosynthetic metabolism (non-stomatal limitation) caused by water stress [75,76,77]. Consistent with previous studies [20,78], stomatal closure (reduced in *g*_s_) effectively minimizes water loss (decrease in *E*), yet simultaneously restricts CO_2_ diffusion into leaves, leading to reduced net photosynthesis (*A*) under moderately salinity levels [79,80]. In this experiment, alfalfa enhanced water-use efficiency (both *W*_g_ and *W*_i_) to cope with water limitation under low-salinity conditions (S1 and S2), a response absent under high-salinity conditions (S3 and S4). Other studies have demonstrated that severe salinity induces biochemical or metabolic (non-stomatal) limitations, such as impaired PSII electron transport or damage to light-harvesting complexes due to reactive oxygen species accumulation [17,76,81,82].

Studies have reported that salinity stress could lead to photoinhibition, which induces photo-oxidative damage to the photosynthetic apparatus and regulation ability [70,83]. Chlorophyll fluorescence measurements (e.g., F_V_/F_M_), which reflect the maximal efficiency of excitation energy capture by “open” PSII reaction centers, show that decreases in this parameter indicate that plants are subjected to photoinhibition [53]. In this experiment, biochar application significantly enhanced the values of F_V_/F_M_ in alfalfa under salinity conditions (Figure 2f and Figure 7), consistent with findings from multiple studies [73,84,85]. This suggests that reduced damage to PSII oxygen-evolving core complexes and reaction centers in alfalfa leaves, potentially resulting from biochar amendment, has the capacity to improve root-zone water status and alleviate the physiological drought stress induced by salinity [76,82]. The concentration of leaf photosynthetic pigments is a critical component of the light-harvesting system, and salinity stress inhibits key enzymes in chlorophyll biosynthesis, such as 5-aminolevulinic acid, which could decrease chlorophyll content [86,87]. In this study, chlorophyll a (Chl a) content, chlorophyll b (Chl b) content, total chlorophyll (total Chl) content, and the ratio of chlorophyll a to chlorophyll b (Chl a:b ratio) significantly decreased under salinity conations (Table 3 and Figure 3), which is consistent with the results of some previous studies [19,38,44]. For instance, Jabborova et al. (2023) [64] observed that reduced leaf chlorophyll contents and carotenoid contents in maize, amaranth, and alfalfa under salinity stress were significantly restored by biochar application. In this experiment, we revealed a significant positive correlation between *A* and chlorophyll content (Figure 7), indicating that biochar enhances photosynthetic pigment levels, thereby promoting CO_2_ assimilation in alfalfa leaves and improving the regulation of gas exchange under salinity stress [88,89]. Meanwhile, biochar application mitigated chlorophyll content loss, potentially preventing impairment of active photosynthetic processes and reducing chlorosis and premature leaf senescence under saline conditions [17,32,34].

### 4.2. Effects of Wheat Straw Biochar Amendment on the Physiological and Biochemical Indices of Alfalfa Under Salinity Stress

Salinity stress induces excessive production of reactive oxygen species (ROS), including superoxide anions (O_2_^−^), hydrogen peroxide (H_2_O_2_), and hydroxyl radicals (OH^−^), which inflict oxidative damage on plant cells [57,90]. Antioxidant enzymes such as dismutase (SOD), catalase (CAT), and peroxidase (POD) are pivotal in scavenging ROS and maintaining redox homeostasis [22,44,91,92]. In this study, elevated levels of H_2_O_2_ and malondialdehyde (MDA) under salinity indicated heightened oxidative stress (Table 3 and Figure 4). Conversely, biochar amendment significantly reduced H_2_O_2_ and MDA concentrations (Figure 4), demonstrating its efficacy in mitigating oxidative damage. Notably, antioxidant enzyme activities (SOD, CAT, and POD) decreased under salt stress with biochar application, which may reflect diminished ROS production owing to improved water availability and ionic balance in the rhizosphere, consistent with findings from prior research [93,94]. Osmotic adjustment represents another critical mechanism through which plants alleviate salt-induced dehydration [17,19,22,57]. Alfalfa accumulated proline and soluble sugar under salinity to maintain osmotic equilibrium and safeguard cellular functions [44]. By contrast, biochar-treated plants exhibited significant proline and soluble sugar levels under salinity conditions (Figure 5d,e), suggesting that improved soil water status reduced the requirement for osmolyte accumulation [6,24]. Thus, biochar mitigates physiological drought stress and diminishes reliance on osmotic protection mechanisms.

Phytohormone regulation also plays a fundamental role in salinity stress adaption [33,63,93]. In this study, indole-3-acetic acid (IAA_leaf_) and gibberellin (GA_leaf_) concentrations decreased under salinity but increased significantly with biochar treatment (Figure 6a,b), indicating restoration of phytohormone homeostasis [95]. These phytohormones are known to promote cell elongation and division, thereby contributing to enhanced growth performance [96,97]. Moreover, IAA_leaf_ and GA_leaf_ levels were negatively correlated with oxidative stress markers (H_2_O_2_ and MDA), while abscisic acid (ABA_leaf_) levels showed a positive correlation (Figure 7). Under salt stress, ABA_leaf_ accumulation drives stomatal closure, enhancing stress tolerance but concurrently restricting growth [18,77]. Biochar significantly reduced ABA_leaf_ content in alfalfa under salinity, potentially by improving soil water availability and downregulating ABA_leaf_ biosynthesis or signaling pathways (e.g., PYL, SAPK2), as proposed in prior studies [33].

In line with our hypothesis, while wheat straw biochar exhibited beneficial effects under salinity stress, excessive application (50 g kg^−1^) weakened its positive impact, which is similar to the results of numerous studies [17,32,43]. This phenomenon may be attributed to the modifications in soil physicochemical properties [24,34]. High biochar application rates, particularly those with alkaline pH arising from high-temperature pyrolysis (>500 °C), may disturb rhizosphere microenvironments, reduce nutrient bioavailability, and alter soil wettability, thereby causing uneven water distribution and reduced photosynthetic activity [36,40]. In this study, wheat straw-derived biochar produced at 500 °C exhibited slight hydrophobicity (Table 1), which may have impaired water uptake under high dosage conditions [23,28,30]. These findings underscore the importance of optimizing biochar application dose.

Although our pot experiment demonstrated that biochar application is effective in alleviating salt stress in alfalfa, it is acknowledged that the pot experiment may not fully replicate field conditions. Environmental factors such as climate, soil texture, and pH vary across regions, while biochar properties differ inherently depending on feedstock and production protocols [87,98,99]. Moreover, crop species and cultivars may exhibit divergent responses to biochar amendment [64,100]. Therefore, field trials across diverse agroecosystems are essential to validate and refine these findings.

## 5. Conclusions

Overall, salinity stress significantly reduced the growth and physiological function of alfalfa (*Medicago sativa* L.) seedlings, triggering a cascade of stress responses as salinity levels increased. At 100 mM NaCl, shoot, root, and total biomass declined by over 70%, while gas exchange traits (*A*, *g*_s_, and *E*) dropped by 58–63%. These changes were accompanied by reductions in F_v_/F_M_ and chlorophyll content, and increases in ABA, H_2_O_2_, MDA, and osmolytes, reflecting oxidative and hormonal stress responses. Wheat straw biochar amendment, particularly at 25 g kg^−1^, effectively mitigated these effects by improving multiple physiological and biochemical attributes. This is shown by relatively stable F_v_/F_M_ and pigment contents; *A*, *g*_s_, and *E* increasing by 28%, 32%, and 27%, respectively; and total biomass improving by 25%. These results suggest that 25 g kg^−1^ biochar is a suitable amendment rate for alleviating moderate salinity stress in alfalfa, through coordinated regulation of physiological, biochemical, and hormonal responses. This highlights its practical suitability as a soil amendment for sustainable alfalfa production in salt-affected soils. From a practical perspective, wheat straw biochar can be produced locally at relatively low cost using agricultural residues, offering a potentially affordable and sustainable strategy for improving pasture productivity in salt-affected regions. Future work should include multi-season field trials to confirm the efficacy of this dosage across varying saline-alkaline soil types and climatic conditions.

## Figures and Tables

**Table 1:** Physicochemical properties of the experimental biochar, bulk soil (B0 treatment), and wheat straw biochar-amended soils (B1 and B2 treatments) used in the study. B0, B1 and B2 refer to 0 g kg^−1^, 25 g kg^−1^, and 50 g kg^−1^ biochar amendment levels, respectively. Values are presented as the arithmetic mean of five replicates (n = 5).

Factor	Biochar	B0-Soil	B1-Soil	B2-Soil
Total C, %	57.59	1.93	7.30	18.95
Total N, %	1.33	0.04	0.39	0.67
Available N, ppm	102.87	62.45	77.71	82.75
Available P, ppm	108.45	40.67	68.63	77.42
Available K, ppm	529.76	114.58	229.91	351.40
pH	8.87	0.31	0.82	1.05
EC, dS m^−1^	2.23	309.01	817.33	1017.39
CEC, cmol + kg^−1^	12.08	5.44	7.76	9.44
Surface area, m^2^ g^−1^	24.59	-	-	-
Total ash, %	30.87	-	-	-
Volatile matter, %	18.82	-	-	-
C stability, %	89.96	-	-	-

**Table 2:** Effects of salinity stress (S) and wheat straw biochar amendment (B) treatment on plant height, stem diameter, leaf area, total biomass, and root/shoot ratio in alfalfa seedlings. Two-way ANOVA results for salinity stress (S), wheat straw biochar amendment (B), and their interaction (S × B) are shown (*F*-values and *p*-values). Different lowercase letters within columns indicate significant differences (*p* ≤ 0.05) between treatments. S0, S1, S2, S3, and S4 refer to 0 mM, 25 mM, 50 mM, 75 mM, and 100 mM NaCl dose levels, respectively. B0, B1, and B2 refer to 0 g kg^−1^, 25 g kg^−1^, and 50 g kg^−1^ wheat straw biochar amendment levels, respectively. Levels of significance are indicated as: n.s = not significant, * = *p* ≤ 0.05, ** = *p* ≤ 0.01, and *** = *p* ≤ 0.001. Data are presented as arithmetic mean ± standard error (n = 5).

Treatment		Plant Height	Stem Diameter	Leaf Area	Total Biomass	Root/Shoot Ratio
		(cm)	(mm)	(cm^2^)	(g pot^−1^)	(None)
B0	S0	24.64 ± 1.57 cd	1.85 ± 0.02 bc	1079.31 ± 77.21 de	17.14 ± 1.66 d	0.39 ± 0.03 de
	S1	23.29 ± 1.20 cde	1.79 ± 0.03 cd	989.07 ± 83.86 ef	15.34 ± 1.14 e	0.38 ± 0.03 efg
	S2	19.19 ± 1.10 f	1.72 ± 0.03 ef	853.82 ± 58.99 g	12.56 ± 0.86 f	0.42 ± 0.05 cd
	S3	13.84 ± 1.24 i	1.61 ± 0.06 g	685.34 ± 47.65 h	8.11 ± 0.61 h	0.48 ± 0.08 b
	S4	11.53 ± 1.18 j	1.47 ± 0.07 h	507.83 ± 43.70 i	3.89 ± 0.45 j	0.53 ± 0.08 a
B1	S0	32.32 ± 1.96 a	1.93 ± 0.05 a	1387.96 ± 46.99 a	23.23 ± 1.58 a	0.30 ± 0.03 j
	S1	28.37 ± 0.82 b	1.92 ± 0.05 a	1284.07 ± 54.51 ab	19.61 ± 1.25 b	0.32 ± 0.02 hi
	S2	24.19 ± 0.98 cd	1.87 ± 0.02 ab	1104.20 ± 53.12 cde	17.69 ± 1.02 cd	0.33 ± 0.04 gh
	S3	21.46 ± 1.64 e	1.79 ± 0.03 cd	981.35 ± 74.44 ef	11.61 ± 1.69 fg	0.37 ± 0.03 efg
	S4	16.45 ± 0.83 gh	1.71 ± 0.04 ef	714.24 ± 58.23 h	6.29 ± 0.49 i	0.44 ± 0.05 bc
B2	S0	27.76 ± 1.23 b	1.90 ± 0.05 ab	1220.01 ± 89.17 bc	19.06 ± 1.81 bc	0.32 ± 0.06 gh
	S1	25.42 ± 1.47 c	1.85 ± 0.05 b	1154.83 ± 74.67 cd	17.28 ± 0.88 d	0.33 ± 0.04 fgh
	S2	22.67 ± 1.52 de	1.76 ± 0.06 de	993.72 ± 64.86 ef	14.86 ± 1.45 e	0.34 ± 0.03 fgh
	S3	16.95 ± 0.95 g	1.70 ± 0.02 f	880.57 ± 52.69 fg	10.46 ± 0.56 g	0.39 ± 0.04 de
	S4	14.24 ± 0.76 hi	1.62 ± 0.04 g	643.92 ± 61.85 h	5.47 ± 0.48 i	0.46 ± 0.01 bc
Significance						
				
Salinity stress (S)		F = 314.95 ***	F = 102.54 ***	F = 211.44 ***	F = 383.06 ***	F = 27.31 ***
Biochar (B)		F = 143.42 ***	F = 175.24 ***	F = 112.23 ***	F = 84.52 ***	F = 28.77 ***
S × B		F = 2.16 *	F = 2.47 **	F = 0.76 n.s	F = 2.46 *	F = 0.32 n.s

**Table 3:** Two-way ANOVA results for salinity stress (S), wheat straw biochar amendment (B), and their interaction (S × B) on plant physiological and biochemical parameters in alfalfa seedlings. *F*-values and *p*-values are shown for shoot biomass, root biomass, leaf net CO_2_ assimilation rate (*A*), stomatal conductance (*g*_s_), transpiration rate (*E*), intrinsic water-use efficiency (*W*_g_), instantaneous water-use efficiency (*W*_i_), maximum quantum efficiency of Photosystem II (Fv/F_M_), chlorophyll content (chlorophyll a: Chl a, chlorophyll b: Chl b, total chlorophyll: total Chl, and the ratio of chlorophyll a to chlorophyll b: Chl a:b ratio), malondialdehyde (MDA) content, hydrogen peroxide (H_2_O_2_) content, catalase (CAT) activity, peroxidase (POD) activity, superoxide dismutase (SOD) activity, proline content, soluble sugar content, indoleacetic acid (IAA_leaf_) content, gibberellic acid (GA_leaf_) content, and abscisic acid (ABA_leaf_) content. Levels of significance are indicated as: n.s = not significant. Data are reported as arithmetic mean ± standard error (n = 5).

	Salinity Stress (S)			Biochar (B)			S × B		
	*df*	*F*	*p*	*df*	*F*	*p*	*df*	*F*	*p*
Shoot biomass	4	274.71	<0.001	2	72.76	<0.001	8	2.21	<0.05
Root biomass	4	318.95	<0.001	2	28.83	<0.001	8	2.18	<0.05
*A*	4	165.81	<0.001	2	129.04	<0.001	8	2.02	<0.05
*g*_s_	4	423.67	<0.001	2	34.21	<0.001	8	2.57	<0.01
*E*	4	1107.54	<0.001	2	82.39	<0.001	8	4.81	<0.001
*W*_g_	4	21.16	<0.001	2	63.31	<0.001	8	3.10	<0.01
*W*_i_	4	9.39	<0.001	2	55.75	<0.001	8	2.33	<0.05
F_V_/F_M_	4	318.77	<0.001	2	20.48	<0.001	8	2.73	<0.01
Chl a content	4	144.54	<0.001	2	116.23	<0.001	8	0.96	n.s
Chl b content	4	88.41	<0.001	2	66.93	<0.001	8	1.10	n.s
Total Chl content	4	151.11	<0.001	2	119.78	<0.001	8	2.06	<0.05
Chl a:b ratio	4	13.02	<0.001	2	12.27	<0.001	8	0.61	n.s
H_2_O_2_ content	4	1498.89	<0.001	2	121.72	<0.001	8	24.72	<0.001
MDA content	4	346.54	<0.001	2	112.68	<0.001	8	4.54	<0.001
CAT activity	4	681.76	<0.001	2	186.34	<0.001	8	24.28	<0.001
POD activity	4	1368.53	<0.001	2	149.13	<0.001	8	30.61	<0.001
SOD activity	4	1115.06	<0.001	2	191.94	<0.001	8	20.64	<0.001
Proline content	4	136.76	<0.001	2	87.84	<0.001	8	2.09	<0.05
Soluble sugar content	4	1661.12	<0.001	2	395.89	<0.001	8	87.17	<0.001
IAA_leaf_ content	4	662.06	<0.001	2	74.16	<0.001	8	3.16	<0.01
GA_leaf_ content	4	813.47	<0.001	2	144.74	<0.001	8	2.55	<0.01
ABA_leaf_ content	4	4830.93	<0.001	2	244.09	<0.001	8	60.65	<0.001

## Data Availability

The data that support the findings of this study are available from the corresponding author upon reasonable request.

## Supplementary Materials

The following supporting information can be downloaded at: https://www.mdpi.com/article/10.3390/plants14131954/s1. References [101,102,103,104,105,106,107,108,109,110,111,112,113,114] are cited in the Supplementary Materials.
