# Supplementary material for: Wheat Straw Biochar Amendment Increases Salinity Stress Tolerance in Alfalfa Seedlings by Modulating Physiological and Biochemical Responses
Source: Plants (Basel). 2025 Jun 26;14(13):1954. doi: 10.3390/plants14131954 (PMC12251899; doi:10.3390/plants14131954)
Supplement: Supplementary file 1 [file plants-14-01954-s001.zip › plants-3686660-supplementary.pdf]

Wheat straw biochar amendment increases salinity stress tolerance in alfalfa seedlings by modulating physiological and biochemical responses

Shangzhi Zhong[1, 2], Pengxin Hou[1, 2], Congcong Zheng[3, 4], Xuechen Yang[5], QiboTao[1, 2], and Juan Sun[1, 2 *]

[1] College of Grassland Science, Qingdao Agricultural University, Qingdao 266109, Shandong, China

[2] Shandong Key Laboratory for Germplasm Innovation of Saline-alkaline Tolerant Grasses and Trees, Qingdao Agricultural University, Qingdao 266109, Shandong, China

[3] Institute of Computing Technology, Chinese Academy of Sciences, Beijing 100190, China

[4] INRAE, UMR, ISPA, 33140, Villenave d'Ornon, France

[5] State Key Laboratory of Ecological Safety and Sustainable Development in Arid Lands, Xinjiang Institute of Ecology and Geography, Chinese Academy of Sciences, Urumqi 830011, China

***Corresponding authors**

Juan Sun

Shandong Key Laboratory for Germplasm Innovation of Saline-alkaline Tolerant Grasses and Trees, College of Grassland Science, Qingdao Agricultural University, Qingdao 266109, Shandong, China

E-mail address: sunjuan@qau.edu.cn

Table S1. Comparison of studies that reported the effects of biochar amendment crop growth in saline soils from both pot and field experiments.

Saline land location	Latitude and longitude	Type of experiment	Target crops	Biochar raw material source	Biochar application rate	Optimal application rate	Effect	References
Wadi El-Assiuty, Assiut, Egypt	27°16'N 31°34'E	Pot	*Raphanus sativus* L.	Wood chips	0%、2.5%、5% (w/w)	2.50%	2.5%: total biomass +142.7% 5%: total biomass -33%	[1]
Dongying, Shandong, China	37°45'N 118°59'E	Pot	*Sesbania cannabina (Retz.)* Poir.	Spartina alterniflora straw	0%、0.5%、1.5%、3% (w/w)	3%	3%: total biomass +141%	[2]
Islamabad, Pakistan	33°39'N 73°9'E	Pot	*Chenopodium quinoa* Willd.	Cotton shells	0%、1%、2% (w/w)	2%	2%: total biomass +60%	[3]
Inner Mongolia Autonomous Region, China.	40°10'N 106°25'E	Pot	*Zea mays* L.	Maize straw	0%、1%、2.5%、5%、10% (w/w)	2.5-5% (3.16%)	2.5%: total biomass +150%	[4]
Multan, Pakistan	30°12'N 71°28'E	Pot	*Triticum aestivum* L.	Rice straw	0、3%、5% (w/w)	5%	5%: total biomass +28%	[5]
Dongying, Shandong, China	37°45'N 118°59'E	Pot	*Suaeda salsa* (L.) Pall.	Wheat straw、Maize straw、Peanut Shell	5、10、20 g/kg	10 g/kg (1%)	10 g/kg (1%): total biomass +115% 20 g/kg (2%): total biomass +102%	[6]
Sheyang, Jiangsu, China	33°46'N 120°15'E	Pot	*Triticum aestivum* L.	Wheat straw	0、15.7、31.4、62.8、94.2、125.6、157.0 g/pot	62.8 g/pot	62.8 g/pot: total biomass +19.4% 157.0 g/pot total biomass -49.5%	[7]

Nanjing, Jiangsu, China	31°57'N 118°50'E	Pot	*Lycopersicon esculentum* Mill.	Wheat straw	0%、2%、4%、8% (w/w)	8%	8%: total biomass +29.5%.	[8]
Yancheng, Jiangsu, China	32°59'N 120°47'E	Field	*Glycine max* L.、*Triticum aestivum* L.	Maize straw	0、3.2、16、32 t/ha	16 t/ha	16 t/ha: wheat grain yield +31.8% Soybean grain yield +23.1%	[9]
Rudong, Jiangsu, China	32°12'N 120°42'E	Field	*Oryza sativa* L.	Wheat straw	0、32、79 t/ha	32 t/ha	32 t/ha: grain yield +11.0% 79 t/ha: grain yield -1.6%	[10]
Sandaweel, Sohage, Egypt	26°33'N 31°42'E	Field	*Triticum aestivum* L.	Corn cob	0、4.8 t/ha	4.8 t/ha	4.8 t/ha grain yield +13.8%	[11]
Dongtai, Jiangsu, China	32°38'N 120°52'E	Field	*Zea mays* L.、*Triticum aestivum* L.	Wheat straw	0、7.5、15、30 t/ha	15 t/ha	15 t/ha: maize grain yield +83.3% 30 t/ha: maize grain yield +80.4%	[12]
Dongtai, Jiangsu, China	32°38'N 120°52'E	Field	*Zea mays* L.、*Triticum aestivum* L.	Wheat straw	0、7.5、15、30 t/ha	15 t/ha	15 t/ha: barley grain yield +59.3% 30 t/ha: barley grain yield +111.7%	[12]
Inner Mongolia Autonomous Region, China.	40°14'N 110°51'E	Field	*Zea mays* L.	Cotton straw, peanut shell, and sawdust	0、30、75 、150 t/ha	150 t/ha	150t/ha: grain yield +347.1%	[13]
Kurna, Bangladesh	22.84°N 89.54°E	Field	*Solanum lycopersicum* L.	Charcoal and agricultural byproducts	1、1.5、2 t/ha	2 t/ha	2 t/ha: grain yield +314.7%	[14]

Table S2. Effects of varying salinity stress (S) and wheat straw biochar amendment (B) treatments on soil available nitrogen (N), available phosphorus (P), available potassium (K), pH and water holding capacity (WHC) in alfalfa-growing systems. Two-way ANOVA results for salinity stress (S), wheat straw biochar amendment (B), and their interaction (S × B) are shown (*F*-values and *P*-values). Different lowercase letters within columns indicate significant differences ($P < 0.05$) among treatments. S0, S1, S2, S3 and S4 refer to 0 mM, 25 Mm, 50 mM, 75 mM, and 100 mM NaCl doses levels, respectively. B0, B1 and B2 refer to 0 g kg $^{-1}$, 25 g kg $^{-1}$, and 50 g kg $^{-1}$ wheat straw biochar amendment levels, respectively. Levels of significance are indicated as: n. s = not significant, * = $P < 0.05$, ** = $P < 0.01$, and *** = $P < 0.001$. Data are presented as arithmetic mean ± standard error **(n = 5)**.

Treatment		Available N ppm	Available P ppm	Available K ppm	pH (none)	WHC mL kg^{-1}
	S0	163.09±4.39 gh	97.31±6.22 hi	334.52±14.58 f	7.59±0.21 efg	107.14±4.51 gh
	S1	177.34±8.54 ef	108.04±2.30 g	310.68±16.48 f	7.42±0.43 fg	105.06±2.73 ghi
B0	S2	189.52±11.12 de	121.10±3.96 f	303.04±19.97 f	7.34±0.37 fg	103.06±3.33 hi
	S3	221.28±8.37 b	165.47±9.68 bc	299.16±7.37 f	7.22±0.31 gh	100.68±9.33 ij
	S4	244.71±15.20 a	180.46±19.07 a	287.98±19.06 f	7.19±0.20 h	96.73±6.06 j
	S0	137.36±7.82 j	78.10±3.19 k	577.69±35.89 bc	8.18±0.15 ab	122.12±3.91 bcd
	S1	152.71±11.82 hi	90.13±3.79 ij	531.95±24.70 cd	7.98±0.32 cd	119.20±5.30 cde
B1	S2	169.85±13.36 fg	102.07±2.94 gh	514.45±17.26 de	7.79±0.32 def	116.09±4.00 de
	S3	199.28±7.86 cd	134.34±7.89 e	508.41±29.96 de	7.67±0.13 efg	111.50±8.50 fg
	S4	233.56±16.50 b	157.41±8.96 c	481.88±40.88 e	7.59±0.38 efg	106.09±3.48 gh
	S0	143.10±4.17 ij	83.26±5.36 jk	651.32±32.77 a	8.35±0.31 a	137.23±5.89 a
	S1	157.22±6.65 gh	95.17±4.54 hi	610.59±21.26 ab	8.10±0.33 bc	130.16±5.55 ab
B2	S2	177.51±10.60 ef	107.05±2.58 g	588.20±29.73 b	8.02±0.52 cd	126.04±1.92 bc
	S3	207.26±7.48 c	145.55±9.56 d	566.89±51.16 bc	7.82±0.24 cde	118.85±7.24 cde
	S4	233.56±12.64 ab	170.22±16.30 ab	538.52±75.69 cd	7.72±0.25 def	112.17±11.25 ef
Significance						
Salinity stress (S)		F=170.59 ***	F=251.83 ***	F=13.54 ***	F=6.96 ***	F=18.18 ***
Biochar (B)		F=30.78 ***	F=42.42 ***	F=490.05 ***	F=28.96 ***	F=84.85 ***
S × B		F=0.27 n.s	F=0.64 n.s	F=0.82 n.s	F=0.14 n.s	F=1.15 n.s

Figure S1. Impacts of wheat straw biochar amendment on relative changes in (**a**) soil available nitrogen (RC-available N), (**b**) available phosphorus (RC-available P), (**c**) available potassium (RC-available K), (**d**) pH (RC-pH), and (**e**) water holding capacity (RC-WHC) in alfalfa-cultivated soil. Two-way ANOVA results for salinity stress (S), wheat straw biochar amendment (B), and their interaction (S × B) are shown (F-values and P-values). Different lowercase letters denote significant differences among salinity stress and biochar amendment treatment combinations at the $P < 0.05$ significance level. S0, S1, S2, S3 and S4 refer to 0 mM, 25 Mm, 50 mM, 75 mM, and 100 mM NaCl doses levels, respectively. B0, B1 and B2 refer to 0 g kg $^{-1}$, 25 g kg $^{-1}$, and 50 g kg $^{-1}$ wheat straw biochar amendment levels, respectively. Levels of significance are indicated as: n. s = not significant, * = $P < 0.05$, ** = $P < 0.01$, and *** = $P < 0.001$. Data are presented as arithmetic mean ± standard error **(n = 5)**.

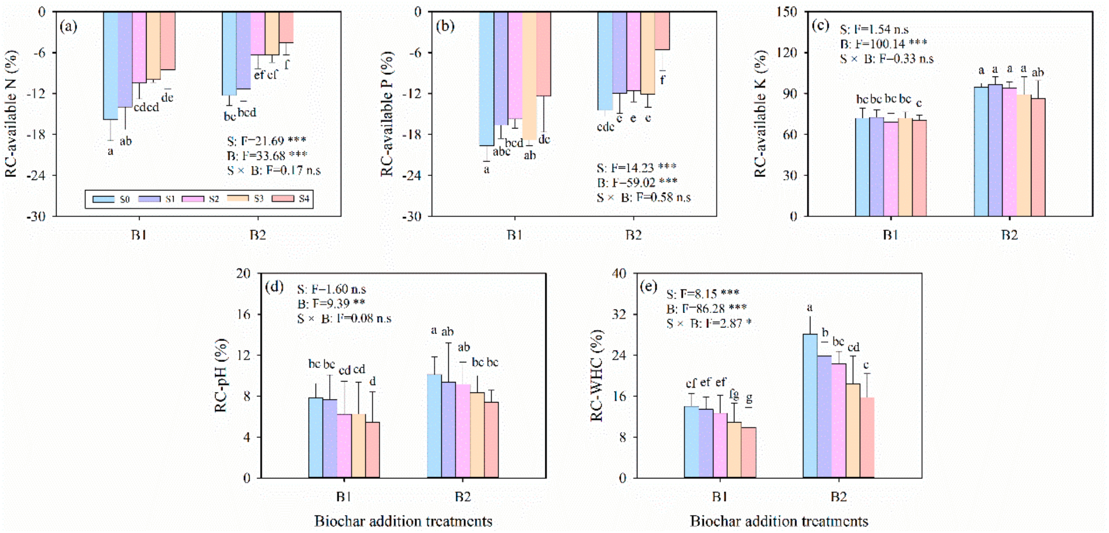

References

[1] Amin, A. E. E. A. Z. (2023). Effects of saline water on soil properties and red radish growth in saline soil as a function of co-applying wood chips biochar with chemical fertilizers. *BMC Plant Biol.* 23 (1), 382. doi: 10.1186/s12870-023-04397-3

[2] Cui, Q., Xia, J., Yang, H., Liu, J., and Shao, P. (2021). Biochar and effective microorganisms promote *Sesbania cannabina* growth and soil quality in the coastal saline-alkali soil of the Yellow River Delta, China. *Sci. Total Environ.* 756, 143801. doi: 10.1016/j.scitotenv.2020.143801

[3] Abbas, G., Abrar, M. M., Naeem, M. A., Siddiqui, M. H., Ali, H. M., Li, Y., et al. (2022). Biochar increases salt tolerance and grain yield of quinoa on saline-sodic soil: multivariate comparison of physiological and oxidative stress attributes. *J Soils Sediment*. 22 (5), 1446-1459. doi: 10.1007/s11368-022-03159-2

[4] Chen, X., Liu, L., Yang, Q., Xu, H., Shen, G., and Chen, Q. (2024). Optimizing Biochar Application Rates to improve soil properties and crop growth in saline–alkali soil. *Sustainability* 16 (6), 2523. doi: 10.3390/su16062523

[5] Abbas, T., Rizwan, M., Ali, S., Adrees, M., Zia-ur-Rehman, M., Qayyum, M. F., et al. (2018). Effect of biochar on alleviation of cadmium toxicity in wheat (*Triticum aestivum* L.) grown on Cd-contaminated saline soil. *Environ. Sci. Pollut. R.* 25, 25668-25680. doi: 10.1007/s11356-017-8987-4

[6] Sun, J., He, F., Shao, H., Zhang, Z., and Xu, G. (2016). Effects of biochar application on *Suaeda salsa* growth and saline soil properties. *Environ. Earth Sci.* 75, 1-6. doi: 10.1007/s12665-016-5440-9

[7] Sun, H., Zhang, H., Shi, W., Zhou, M., and Ma, X. (2019). Effect of biochar on nitrogen use efficiency, grain yield and amino acid content of wheat cultivated on saline soil. *Plant, Soil & Environ.* 65 (2), 83-89. doi: 10.17221/525/2018-PSE

[8] She, D., Sun, X., Gamareldawla, A. H. D., Nazar, E. A., Hu, W., Edith, K., et al. (2018). Benefits of soil biochar amendments to tomato growth under saline water irrigation. *Sci. Rep.* 8 (1), 14743. doi: 10.1038/s41598-018-33040-7

[9] Lin, X., Xie, Z., Zheng, J., Liu, Q., Bei, Q., and Zhu, J. (2015). Effects of biochar application on greenhouse gas emissions, carbon sequestration and crop growth in coastal saline soil. *Eur. J. Soil Sci.* 66 (2), 329-338. doi: 10.1111/ejss.12225

[10] Abulaiti, A., She, D., Liu, Z., Sun, X., and Wang, H. (2023). Application of biochar and polyacrylamide to revitalize coastal saline soil quality to improve rice growth.

Environ. Sci. Pollut. R. 30 (7), 18731-18747. doi: 10.1007/s11356-022-23511-w

[11] El-Sayed, M. E., Hazman, M., Abd El-Rady, A. G., Almas, L., McFarland, M., Shams El Din, A., et al. (2021). Biochar reduces the adverse effect of saline water on soil properties and wheat production profitability. *Agric.* 11 (11), 1112. doi: 10.3390/agriculture11111112

[12] Sun, Y., Yang, J., Yao, R., Chen, X., and Wang, X. (2020). Biochar and fulvic acid amendments mitigate negative effects of coastal saline soil and improve crop yields in a three year field trial. *Sci. Rep.* 10 (1), 8946. doi: 10.1038/s41598-020-65730-6

[13] Yue, Y., Lin, Q., Li, G., Zhao, X., and Chen, H. (2023). Biochar Amends saline soil and enhances maize growth: three-year field experiment findings. *Agron*. 13 (4), 1111. doi: 10.3390/agronomy13041111

[14] Zonayet, M., Paul, A. K., Faisal-E-Alam, M., Syfullah, K., Castanho, R. A., and Meyer, D. (2023). Impact of biochar as a soil conditioner to improve the soil properties of saline soil and productivity of tomato. *Sustainability* 15 (6), 4832. doi: 10.3390/su15064832
